# Performance of Computer‐Aided Detection Software in Tuberculosis Case Finding in Township Health Centers in China

**DOI:** 10.1002/cdt3.70001

**Published:** 2025-04-02

**Authors:** Xuefang Cao, Boxuan Feng, Bin Zhang, Dakuan Wang, Jiang Du, Yijun He, Tonglei Guo, Shouguo Pan, Zisen Liu, Jiaoxia Yan, Qi Jin, Lei Gao, Henan Xin

**Affiliations:** ^1^ NHC Key Laboratory of Systems Biology of Pathogens, National Institute of Pathogen Biology, and Center for Tuberculosis Research Chinese Academy of Medical Sciences and Peking Union Medical College Beijing China; ^2^ Key Laboratory of Pathogen Infection Prevention and Control (Ministry of Education), National Institute of Pathogen Biology Chinese Academy of Medical Sciences & Peking Union Medical College Beijing China; ^3^ Center for Disease Control and Prevention of Zhongmu County Zhengzhou Henan China

**Keywords:** artificial intelligence, case finding, chest X‐ray, computer‐aided detection, tuberculosis

## Abstract

**Background:**

Computer‐aided detection (CAD) software has been introduced to automatically interpret digital chest X‐rays. This study aimed to evaluate the performance of CAD software (JF CXR‐1 v3.0, which was developed by a domestic Hi‐tech enterprise) in tuberculosis (TB) case finding in China.

**Methods:**

In 2019, we conducted an internal evaluation of the performance of JF CXR‐1 v3.0 by reading standard images annotated by a panel of experts. In 2020, using the reading results of chest X‐rays by a panel of experts as the reference standard, we conducted an on‐site prospective study to evaluate the performance of JF CXR‐1 v3.0 and local radiologists in TB case finding in 13 township health centers in Zhongmu County, Henan Province.

**Results:**

Internal assessment results based on 277 standard images showed that JF CXR‐1 v3.0 had a sensitivity of 85.94% (95% confidence interval [CI]: 77.42%, 94.45%) and a specificity of 74.65% (95% CI: 68.81%, 80.49%) to distinguish active TB from other imaging conditions. In the on‐site evaluation phase, images from 3705 outpatients who underwent chest X‐ray detection were read by JF CXR‐1 v3.0 and local radiologists in parallel. The imaging diagnosis of local radiologists for active TB had a sensitivity of 32.89% (95% CI: 22.33%, 43.46%) and a specificity of 99.28% (95% CI: 99.01%, 99.56%), while JF CXR‐1 v3.0 showed a significantly higher sensitivity of 92.11% (95% CI: 86.04%, 98.17%) (*p* < 0.05) and maintained high specificity at 94.54% (95% CI: 93.81%, 95.28%).

**Conclusions:**

CAD software could play a positive role in improving the TB case finding capability of township health centers.

## Summary

- CAD software might be applied to help human readers improve overall TB diagnosis in primary health center, especially in resource‐limited areas with high TB burden.
- Compared with local radiologists, the diagnosis of JF CXR‐1 v3.0 for active TB had a significantly higher sensitivity and a similar specificity.

## Graphical abstract

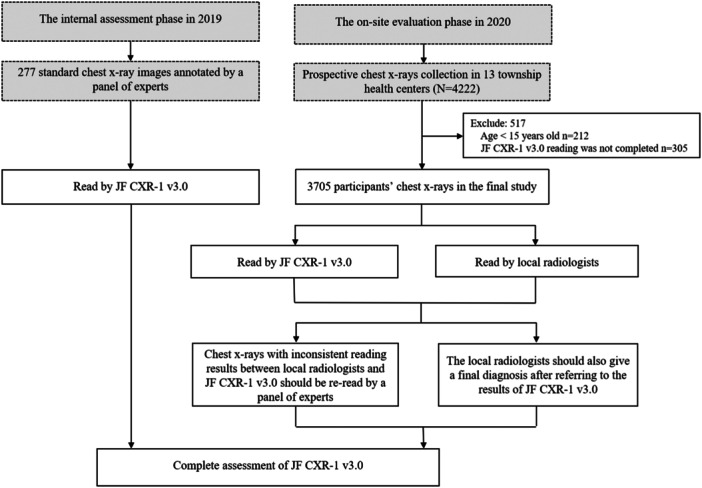

## 1 Introduction

Tuberculosis (TB) remains one of the major infectious diseases that seriously endangers human health, with an estimate of 10 million new cases annually worldwide. Moreover, there are still wide gaps between the estimated number of people who develop TB each year (incident cases) and the number of people newly diagnosed and officially reported as a TB case [1]. Intensified efforts should be made to close the gaps between incidence and reporting, and strengthening active TB case screening to improve TB diagnosis and treatment is important to ensure that people are correctly diagnosed and started on the most effective treatment regimen as early as possible. Currently, screening for symptoms, chest X‐ray, and molecular rapid diagnostics have all been recommended as initial screening tools [2]. Symptom screening is feasible, easy to implement, and economical but has low sensitivity. Molecular tests are rapid and sensitive but have major resource implications and require additional equipment. Chest X‐ray is regarded as a good screening tool to identify people in need of further testing to diagnose TB, especially when the follow‐up diagnostic test is expensive [3, 4]. However, one of the challenges is the paucity of professionals to interpret radiographic images in resource‐constrained settings [5] and substantial intra‐ and interreader variation [6, 7].

In recent years, with the development of artificial intelligence (AI) in the medical field, it has been applied to the analysis of radiologic images to quickly evaluate digital chest X‐rays to recognize abnormalities in lung fields referred to as computer‐aided detection (CAD). It was regarded as one potential solution to overcome the personnel shortage and significant scale‐up of TB screening. Several CAD software products have been developed to interpret digital chest X‐rays for abnormalities suggestive of TB or other diseases, such as CAD4TB (Delft Imaging, Netherlands), Lunit INSIGHT CXR (Lunit, South Korea), and JF CXR‐1 (JF HEALTHCARE, China). In 2020, the World Health Organization (WHO) first recommended that CAD has the potential to be used as an alternative to human interpretation of plain digital chest X‐ray for TB screening and triage among individuals aged 15 years and older [8]. However, the WHO ultimately did not recommend specific CAD products as the market is constantly expanding and more evaluation is needed to accumulate evidence. In China, 70% of TB cases occur in rural areas, and radiologists from primary health centers play an important role in accurately identifying abnormal chest X‐ray results suggestive of TB and promptly transferring patients to the local designated medical institutions for TB [9]. Whether CAD performs better than radiologists and whether it can be used to improve the diagnostic ability of primary health centers has rarely been explored. JF CXR‐1 v3.0 is a CAD product based on cutting‐edge deep learning (DL) technology, which was developed by a domestic Hi‐tech enterprise (JF Healthcare, Nanchang, China). It has been trained on approximately 300,000 chest X‐ray images from township level hospitals in multiple provinces across China. JF CXR‐1 v3.0 uses the same internal algorithm as JF CXR‐2 [10, 11]. The difference is that it can diagnose multiple diseases at the same time, including prior TB, pneumonia, or lung nodule/mass, to assist radiologists with differential diagnosis. Each disease is a separate dichotomous model and does not affect the results for TB. Thus, the current study aimed to evaluate the performance of JF CXR‐1 v3.0 on TB case finding by reading standard images and application in primary health centers in China.

## 2 Materials and Methods

### 2.1 Study Design and Population

For the smooth implementation of the on‐site prospective study, an internal assessment was done before the start of the study to give a preliminary assessment of the performance of JF CXR‐1 v3.0. Therefore, this study consisted of two phases. In the internal assessment, 277 standard images were annotated by a panel of experts (composed of one radiologist and two clinical experts with more than 10 years of experience in chest X‐ray reading and TB diagnosis) (Supporting Information S1: Table 1). Then, the standard images were uploaded to JF CXR‐1 v3.0, and abnormality scores ranging from 0 (*normal*) to 1 (*highly abnormal*) were calculated, which may be interpreted as the probability that the subject is suffering from active TB, prior TB, pneumonia, or lung nodule/mass visible on chest X‐ray. A developer‐recommended threshold score of 0.5 was used in the current study.

The second phase was an on‐site prospective study from June to December 2020, aiming to evaluate the performance of JF CXR‐1 v3.0 in TB case finding capability in 13 township health centers in Zhongmu County, Henan Province. The inclusion criteria of the study participants included outpatients who visited the radiology department for chest X‐rays, patients aged 15 years or older, and patients who voluntarily participated in this study. For each study participant, socio‐demographic information was collected by local radiologists, including age, sex, level of education, and smoking status. History of prior TB, history of close contact with TB patients, and reasons for taking chest X‐ray were also collected. In addition, three multiple choice questions were set for the diagnosis of TB and other lung diseases. First, a digital chest X‐ray was performed for each study participant, which was independently read by local radiologists (initial reading) and classified as “normal, active TB, prior TB, pneumonia, lung nodule/mass, or other lesions.” Then, the chest X‐rays were uploaded to JF CXR‐1 v3.0, and continuous abnormality scores were calculated. Scores above the threshold scores of 0.5 should be classified as “active TB, prior TB, pneumonia, lung nodule/mass, or pleural lesions.” Finally, the local radiologists should also give a final diagnosis after referring to the results of JF CXR‐1 v3.0 to evaluate the local radiologists’ acceptance for the results of the JF CXR‐1 v3.0. Besides, chest X‐rays with inconsistent reading results between local radiologists’ initial reading and JF CXR‐1 v3.0 were reread by a panel of experts (composed of one radiologist and two clinical experts with more than 10 years of experience in reading chest X‐rays and TB diagnosis). The positive criterion is that every panel member gave a positive diagnosis.

The suspected active pulmonary TB patients identified by local radiologists and JF CXR‐1 v3.0 were transferred to the Center for Disease Control and Prevention of Zhongmu County, Zhengzhou, Henan, China for further diagnosis, which is the local designated medical institution for TB.

### 2.2 Statistical Analysis

Statistical analyses were performed using SAS version 9.4 (SAS Institute, Cary, NC). The sensitivities and specificities (and exact binomial 95% confidence intervals [CIs]) of local radiologists and JF CXR‐1 v3.0 in the diagnosis of abnormal categories were calculated. In addition, Cohen's kappa coefficient was calculated to evaluate the agreement between local radiologists and JF CXR‐1 v3.0. McNemar's test was used to compare the sensitivities and specificities of local radiologists and JF CXR‐1 v3.0 in the diagnosis of different abnormalities. Pearson's *χ*² test or Fisher's exact test was used to assess the distribution of categorical variables. A two‐tailed *p* value < 0.05 was considered statistically significant.

## 3 Results

### 3.1 Performance of JF CXR‐1 v3.0 at the Internal Assessment Phase

Table 1 shows that JF CXR‐1 v3.0 had a sensitivity of 85.94% (95% CI: 77.42%, 94.45%) and a specificity of 100.00% (95% CI: 100.00%, 100.00%) to differentiate active TB from normal chest X‐rays. When differentiating active TB from other imaging conditions (including normal, pneumonia, prior TB, and lung nodule/mass), the sensitivity remained the same, while the specificity dropped to an acceptable value of 74.65% (95% CI: 68.81%, 80.49%).

**Table 1:** Accuracy of JF CXR‐1 v3.0 in the diagnosis of standard chest X‐ray images at internal assessment phase.

Items	JF CXR‐1 v3.0+/PEs+ (%)	JF CXR‐1 v3.0−/PEs+ (%)	JF CXR‐1 v3.0+/PEs− (%)	JF CXR‐1 v3.0−/PEs− (%)	Sensitivity (%) (95% CI)	Specificity (%) (95% CI)
Active TB vs. Normal chest X‐rays^a^	55 (19.86)	9 (6.47)	0 (0.00)	75 (53.96)	85.94 (77.42, 94.45)	100.00 (100.00, 100.00)
Active TB vs. Normal and other lung lesions^b^	55 (19.86)	9 (3.25)	54 (19.49)	159 (57.40)	85.94 (77.42, 94.45)	74.65 (68.81, 80.49)

Abbreviations: CI, confidence interval; PE, panel of expert; TB, tuberculosis.

^a^ JF CXR‐1 v3.0's ability to differentiate active TB from normal chest X‐rays.

^b^ JF CXR‐1 v3.0's ability to differentiate active TB from other imaging conditions (including normal, pneumonia, prior TB, and lung nodule/mass).

### 3.2 Characteristics of the Study Population at the On‐Site Evaluation Phase

**Figure 1:**
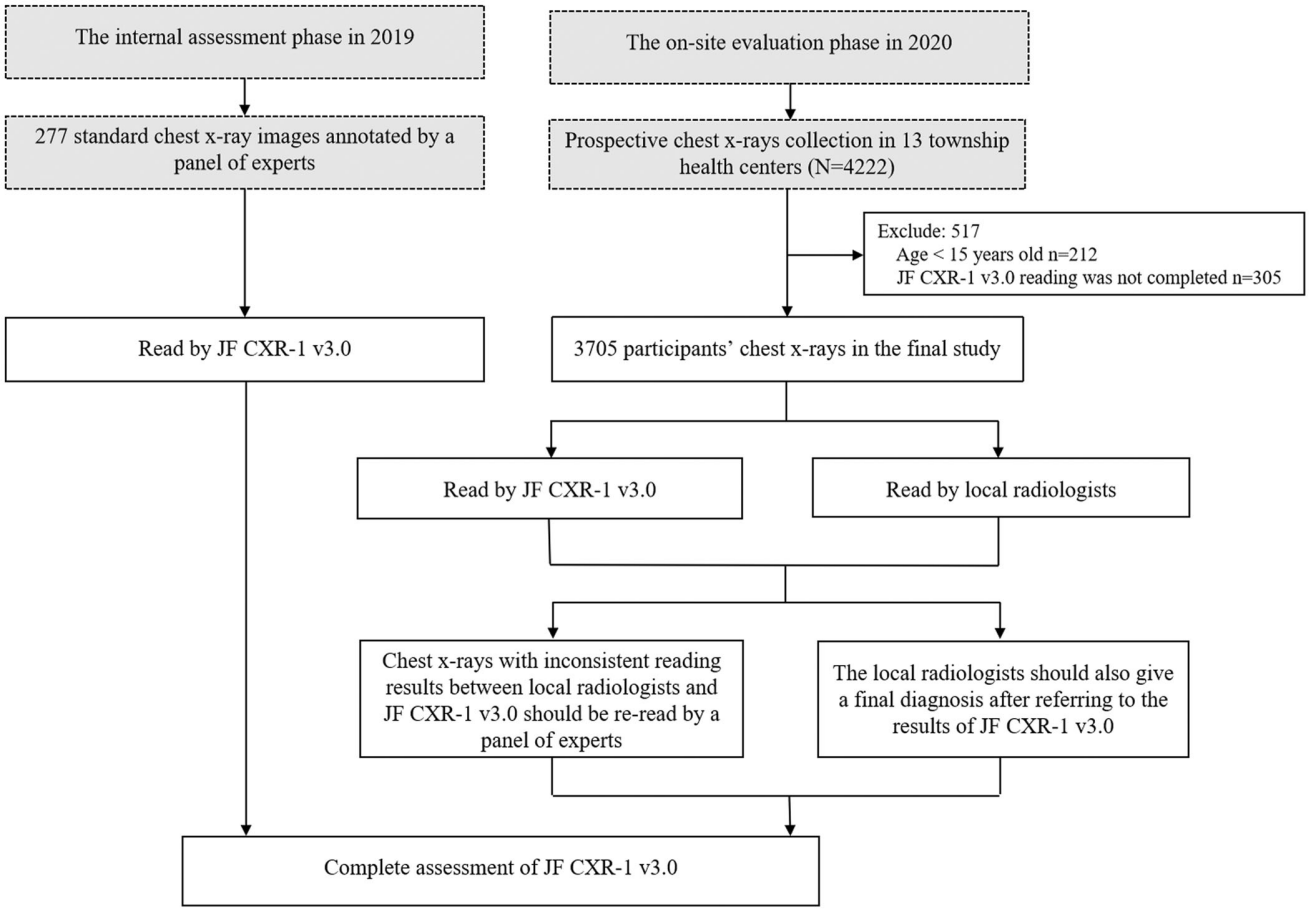
Flowchart of the study implementation. In 2019, 277 standard chest X‐rays with typical abnormalities annotated by a panel of experts were used for internal evaluation of the performance of JF CXR‐1 v3.0. In 2020, an on‐site prospective study was implemented to evaluate the performance of JF CXR‐1 v3.0 in 13 township health centers in Zhongmu County, Henan Province. In total, 3705 subjects were included in the final study. Each chest X‐ray was independently read by local radiologists and JF CXR‐1 v3.0. Chest X‐rays with inconsistent readings between local radiologists and JF CXR‐1 v3.0 were reread by a panel of experts. The local radiologists should also give a final diagnosis after referring to the results of JF CXR‐1 v3.0. Finally, we completed the evaluation of the performance of JF CXR‐1 v3.0.

As shown in Figure 1, 4222 chest X‐rays of participants were prospectively collected, and 3705 participants were included in the final study after excluding patients whose JF CXR‐1 v3.0 reading was not completed or whose age did not meet the criteria of 15 years or older. Table 2 shows the major characteristics of the study participants. Overall, 54.84% (2032/3705) of the participants were male, and 1.73% (64/3705) of participants reported a history of prior TB. Approximately 27.96% (1036/3705) of the participants had respiratory tract symptoms of cough, and 1.49% (55/3704) of the participants reported a history of close contact with TB patients.

**Table 2:** Characteristics of the study population at the on‐site evaluation phase.

Variables^a^	*n* (%)
Sex	
Male	2032 (54.84)
Female	1673 (45.16)
Age (years)	
15–24	284 (7.67)
25–34	568 (15.33)
35–44	309 (8.34)
45–54	756 (20.4)
55–64	724 (19.54)
≥65	1064 (28.72)
Highest education level	
Middle school or lower	3039 (83.12)
High school	416 (11.38)
College or higher	201 (5.74)
Smoking status	
Never smoked	2763 (74.57)
Ever smoked	942 (25.43)
History of prior TB	
No	3641 (98.27)
Yes	64 (1.73)
History of close contact with TB patients	
No	3639 (98.51**)**
Yes	55 (1.49)
Reasons for taking chest X‐ray	
Fever	
No	3659 (98.76)
Yes	46 (1.24)
Cough of any duration	
No	2669 (72.04)
Yes	1036 (27.96)
Hemoptysis	
No	3691 (99.62)
Yes	14 (0.38)
Short of breath	
No	3168 (85.51)
Yes	537(14.49)
Nights	
No	3698 (99.81)
Yes	7 (0.19)
Weight loss	
No	3696 (99.76)
Yes	9 (0.24)

Abbreviation: TB, tuberculosis.

^a^ Data might not sum to the total because of missing data.

### 3.3 Performance Evaluation of Local Radiologists for Initial Reading and JF CXR‐1 v3.0 to Detect Abnormalities on Chest X‐Rays at the On‐Site Evaluation Phase

The comparable readings between local radiologists and JF CXR‐1 v3.0 are summarized in Supporting Information S1: Table 2. For active TB, the concordance between local radiologists and JF CXR‐1 v3.0 was 93.65% with a *κ* coefficient of 0.25 (95% CI: 0.18, 0.31). As shown in Table 3, taking the reports of the expert panel as a reference, the imaging diagnosis of local radiologists for active TB had a sensitivity of 32.89% (95% CI: 22.33%, 43.46%) and a specificity of 99.28% (95% CI: 99.01%, 99.56%), while the diagnosis of JF CXR‐1 v3.0 showed a higher sensitivity of 92.11% (95% CI: 86.04%, 98.17%) (*p* < 0.05) and maintained high specificity at 94.54% (95% CI: 93.81%, 95.28%) (*p* < 0.05).

**Table 3:** Performance evaluation of local radiologists for initial reading and JF CXR‐1 v3.0 to detect abnormalities on chest X‐rays with the diagnosis of a panel of experts as reference.

Items	LRs+/PEs+ (%)	LRs−/PEs+ (%)	LRs+/PEs− (%)	LRs−/PEs− (%)	Sensitivity (%) (95% CI)	Specificity (%) (95% CI)
Active TB vs. Normal and other lung lesions^a^	25 (0.67)	51 (1.38)	26 (0.70)	3603 (97.25)	32.89 (22.33, 43.46)	99.28 (99.01, 99.56)
Prior TB vs. Normal and other lung lesions^c^	33 (0.89)	44 (1.19)	65 (1.75)	3563 (96.17)	42.86 (31.80, 53.91)	98.21 (97.78, 98.64)
Pneumonia vs. Normal and other lung lesions^d^	126 (3.40)	10 (0.27)	467 (12.60)	3102 (83.72)	92.65 (88.26, 97.03)	86.92 (85.81, 88.02)
Lung nodule/mass vs. Normal and other lung lesions^e^	18 (0.49)	3 (0.08)	36 (0.97)	3648 (98.46)	85.71 (70.75, 100.00)	99.02 (98.71, 99.34)

**Items**	**JF CXR‐1 v3.0** + **/PEs** + **(%)**	**JF CXR‐1 v3.0‐/PEs** + **(%)**	**JF CXR‐1 v3.0** + **/PEs‐ (%)**	**JF CXR‐1 v3.0‐/PEs‐ (%)**	**Sensitivity (%) (95% CI)**	**Specificity (%) (95% CI)**
Active TB vs. Normal and other lung lesions^a^	70 (1.89)	6 (0.16)	198 (5.34)	3431 (92.60)	92.11 (86.04, 98.17)^b^	94.54 (93.81, 95.28)^b^
Prior TB vs. Normal and other lung lesionss^c^	61 (1.65)	16 (0.43)	702 (18.95)	2926 (78.97)	79.22 (70.16, 88.28)^b^	80.65 (79.37, 81.94)^b^
Pneumonia vs. Normal and other lung lesions^d^	134 (3.62)	2 (0.05)	566 (15.28)	3003 (81.05)	98.53 (96.50, 100.00)^b^	84.41 (82.94, 85.34)^b^
Lung nodule/mass vs. Normal and other lung lesions^e^	15 (0.40)	6 (0.16)	96 (2.59)	3588 (96.84)	71.43 (52.11, 90.75)	97.39 (96.88, 97.91)^b^

Abbreviations: CI, confidence interval; LR, local radiologist; PE, panel of experts; TB, tuberculosis.

^a^ The ability to differentiate active TB from other imaging conditions (including normal, pneumonia, prior TB, lung nodule/mass and pleural lesions).

^b^ Compared with the sensitivities and specificities of local radiologists in the diagnosis of different abnormalities by McNemar's test, *p* < 0.05.

^c^ The ability to differentiate prior TB from other imaging conditions (including normal, active TB, pneumonia, lung nodule/mass, and pleural lesions).

^d^ The ability to differentiate pneumonia from other imaging conditions (including normal, active TB, prior TB, lung nodule/mass, and pleural lesions).

^e^ The ability to differentiate lung nodule/mass from other imaging conditions (including normal, active TB, pneumonia, prior TB, and pleural lesions).

As shown in Table 4, specificity was significantly lower in older people (*p* < 0.001), male (*p* = 0.043), participants with a history of prior TB (*p* < 0.001), and participants who ever smoked (*p* = 0.030).

**Table 4:** Associations between characteristics and accuracy for JF CXR‐1 v3.0 in the diagnosis of active TB.

Variables^a^	Sensitivity		Specificity	
	Positive test, *n*/*N* (%)	*p* value	Negative test, *n*/*N* (%)	*p* value
Age		0.273^b^		< 0.001^c^
Young and middle age (15–59 years)	20/23 (86.96)		2197/2245 (97.86)	
Older age (≥ 60 years)	50/53 (94.34)		1234/1384 (89.16)	
Sex		1.000^b^		0.043^c^
Male	45/49 (92.59)		1861/1983 (93.85)	
Female	25/27 (91.84)		1570/1646 (95.38)	
Smoking status		1.000^b^		0.030^c^
Never smoked	48/52 (92.31)		2576/2711 (95.02)	
Ever smoked	22/24 (91.67)		855/918 (93.14)	
History of prior TB		1.000^b^		< 0.001^c^
No	44/48 (91.67)		3415/3593 (95.05)	
Yes	26/28 (92.86)		16/36 (44.44)	

Abbreviation: TB, tuberculosis.

^a^ Data might not sum to the total because of missing data.

^b^ *p* for Fisher's exact test.

^c^ *p* for *χ* ^2^ test.

### 3.4 Local Radiologists’ Acceptance for the Inconsistent Results Between Local Radiologists’ Initial Reading and JF CXR‐1 v3.0 at the On‐Site Evaluation Phase

When the local radiologist disagrees with the JF CXR‐1 v3.0 diagnosis, the local radiologist has the opportunity to change their decision and give a final diagnosis. As shown in Supporting Information S1: Table 3, there were 235 inconsistent results between radiologists and JF CXR‐1 v3.0 in the diagnosis of active TB. For discordant results with LRs+/JF CXR‐1 v3.0−, after referring to the reading results of JF CXR‐1 v3.0, the radiologists accepted 5 AI diagnostic results for chest X‐rays (55.56%, 5/9). However, for discordant results with LRs−/JF CXR‐1 v3.0+, the proportion of radiologists accepting JF CXR‐1 v3.0's diagnostic results was only 13.27% (30/226).

## 4 Discussion

In the present study, compared with local radiologists, the diagnosis of JF CXR‐1 v3.0 for active TB had a significantly higher sensitivity and a similar specificity at both internal assessment and on‐site evaluation phase. This indicates that CAD software might play a supportive role in improving TB case detection capability in primary medical institutions in China.

Qin and colleagues conducted two retrospective evaluation of DL systems, including CAD4TB (v6), InferReadDR (v2), Lunit INSIGHT for Chest Radiography (v4.9.0), JF CXR‐1 (v2), and qXR (v3) to detect TB‐related abnormalities in chest X‐rays and found that all AI systems significantly outperformed human readers [12, 13]. Yang Y and colleagues conducted a prospective multicenter clinical research study in six general infectious disease hospitals across China, which found that compared to the results from radiologists on the board, JF CXR‐1 showed a sensitivity of 94.2% (95% CI: 92.0%–95.8%) and a specificity of 91.2% (95% CI: 88.5%–93.2%) for the diagnosis of TB [14]. Similar findings were observed in the current study, which found that the sensitivity of JF CXR‐1 v3.0 in detecting active TB abnormalities was higher than that of local radiologists (*p* < 0.05) and maintained high specificity simultaneously, which met WHO‐recommended minimal accuracy for pulmonary TB triage tests (90% for sensitivity, 70% for specificity) [15]. However, we found that the sensitivity of local radiologists in detecting active TB abnormalities is relatively low. The main reason might be that local radiologists do not have enough knowledge of TB imaging, which leads to more missed diagnosis of TB patients. Therefore, relevant training should be carried out to strengthen the diagnostic capacity of grassroots radiologists. Besides, unlike the above two studies that used different AI software to read chest X‐rays retrospectively, our study is a prospective study based on the routine work of township health centers, which found that local radiologists’ acceptance for CAD software results is still very limited, especially for positive results given by CAD software. The main reason might be that local radiologists do not have enough confidence in the performance of the software. Therefore, it is urgent to strengthen the scientific evaluation and advocacy training of CAD software, thereby increasing the confidence of human readers for CAD software in the diagnosis of diseases.

We found that the specificity of JF CXR‐1 v3.0 for the detection of active TB was significantly associated with age, sex, smoking status, and history of prior TB, which was consistent with previous findings [13, 16]. In this study, the proportion of males (46.06%, 936/2032) who smoked was significantly higher than that of females (0.35%, 6/1673) (*p* < 0.05). A previous study reported that smoking might lead to diffuse interstitial pulmonary fibrosis [17], which might be misclassified by CAD software, resulting in a lower specificity of AI in detecting active TB on chest X‐rays of males than females. Older people are prone to other lung diseases, such as pneumonia and chronic bronchitis [18], which might affect the accuracy of AI in the diagnosis of active TB. In addition, participants with a history of TB might have abnormalities on their chest X‐ray images (e.g., fibrotic scarring, nodules without calcification), which were not indicative of current TB disease but might lead to false positives in AI diagnosis, thereby affecting the accuracy of the software. Thus, population characteristics might influence the performance of CAD systems. It may be possible to combine demographic data and clinical data with abnormality scores of CAD systems when training the AI algorithms to further improve their algorithms and achieve individualized risk scores in future software iterations.

In the present study, we used the threshold score of 0.5 for JF CXR‐1 v3.0 and acquired both high sensitivity (92.11%) and specificity (94.54%) for detection of active TB. However, a recent study evaluated the performance of the same version of JF CXR‐1 for detecting TB on chest X‐ray images in Ho Chi Minh City, Vietnam, which reported that when matching the sensitivity of 95.5%, the cut‐off threshold was 0.83 and the specificity was only 41.0% [11]. It implied that variation exists in AI performance across different contexts, and the threshold abnormality scores probably need to vary depending on the test population and needs.

In addition, CAD software is suitable for the application in the detection of TB and the prevention treatment of latent tuberculosis infection among key populations in primary areas, which has been emphasized in Technical Specifications for Tuberculosis prevention and control in China (2020 edition) [19]. However, since no such software has been approved for clinical use when we conducted this study, its application was still limited. As recommended by the WHO, several studies used CAD software in combination with other rapid bacteriological tests, such as the Xpert MTB/RIF, to promote its use in TB diagnosis [20, 21, 22]. This “one‐stop shop” clinic might be a setting that can benefit from the use of CAD software, which is considered worth trying.

When interpreting the results of the study, several limitations should be kept in mind. First, due to logistical and budgetary constraints, we did not collect sputum samples from each participant for pathogen detection in our study. Therefore, we used a panel of expert readings as the reference standard instead of using culture‐based methods, which remain recommended by the WHO, as a reference standard for detecting TB [8]. Second, participants in our study were those who required chest X‐rays. Therefore, the sociodemographic characteristics of the participants, the TB epidemic of the study site, the season of research implementation, and other factors may affect the proportion of participants with abnormal TB imaging, which might affect the assessment of the diagnostic accuracy of the software. Third, approximately 7.6% (305/4,010) of participants’ chest X‐rays were not successfully imported into the CAD system for reading, which might also introduce bias to the results. Finally, our research is an observational study, not an interventional study. Therefore, whether the use of CAD software can effectively improve the diagnosis level of radiologists still needs to be evaluated by randomized controlled trials.

In conclusion, our results suggest that automated reading using JF CXR‐1 v3.0 has better performance than that of radiologists in township health centers in China under real‐world conditions in assessing abnormalities indicative of active TB. This provides a clue that CAD software may be applied to help human readers improve overall TB diagnosis, especially in resource‐limited areas with a high TB burden. However, our findings require further validation by observational studies and interventional studies in different regions and target populations.

## Author Contributions

Henan Xin designed the study. Xuefang Cao, Henan Xin, Boxuan Feng, Bin Zhang, Dakuan Wang, Jiang Du, Yijun He, Tonglei Guo, Shouguo Pan, Zisen Liu, and Jiaoxia Yan were in charge of data management. Xuefang Cao and Henan Xin did data analysis and wrote the report. Qi Jin commented on the report and improved English writing. Xuefang Cao, Henan Xin, Bin Zhang, Boxuan Feng, and Dakuan Wang participated in the data interpretation. Xuefang Cao, Jiang Du, Yijun He, Tonglei Guo, Shouguo Pan, Zisen Liu, Bin Zhang, Dakuan Wang, and Jiaoxia Yan organized investigations at the study sites. All authors contributed to review and revision and have seen and approved the final version of the manuscript.

## Ethics Statement

The study protocol was approved by the ethics committees of the Institute of Pathogen Biology, Chinese Academy of Medical Sciences, Beijing, China (approval IPB‐2019‐10).

## Conflicts of Interest

Professor Lei Gao is a member of Chronic Diseases and Translational Medicine editorial board and is not involved in the peer review and decision process of this article. The other authors declare no conflicts of interest.

## Supporting information

Supporting information.

## Data Availability

The data underlying this article will be shared upon a reasonable request to the corresponding author.
