# Supplementary material for: Performance of Computer‐Aided Detection Software in Tuberculosis Case Finding in Township Health Centers in China
Source: Chronic Dis Transl Med. 2025 Apr 2;11(2):140–7. doi: 10.1002/cdt3.70001 (PMC12142701; doi:10.1002/cdt3.70001)
Supplement: Supplementary file 1 — Supporting information. [file CDT3-11-140-s001.docx]

**Supplementary Table 1. Basic information of 277 standard chest** X**-ray images at the internal assessment phase.**

| **Type of lesion** | **Number** | **Form** |
| --- | --- | --- |
| Normal | 75 | DICOM |
| Active TB | 64 | JPG |
| Prior TB | 61 | DICOM |
| Pneumonia | 31 | JPG |
| Lung nodule/mass | 46 | JPG |
| **Total** | 277 | JPG:141，DICOM:136 |

Abbreviation: TB, tuberculosis.

**Supplementary Table 2. The agreement between initial reading of local radiologists and JF CXR-1 v3.0 at the on-site evaluation phase.**

|  | **LRs+ / JF CXR-1 v3.0+**  **(%)** | **LRs- / JF CXR-1 v3.0+**  **(%)** | **LRs+/ JF CXR-1 v3.0-**  **(%)** | **LRs- /JF CXR-1 v3.0 -**  **(%)** | **Kappa**  **(95% CI)** | **Concordant (%)** |
| --- | --- | --- | --- | --- | --- | --- |
| **Active TB** | 42 (1.13) | 226 (6.10) | 9 (0.24) | 3428 (92.52) | 0.25  (0.18, 0.31) | 93.65 |
| **Prior TB** | 82 (2.21) | 681 (18.38) | 16 (0.43) | 2926 (78.97) | 0.15  (0.12, 0.18) | 80.18 |
| **Pneumonia** | 349 (9.42) | 351 (9.47) | 244 (6.59) | 2761 (74.52) | 0.44 (0.41,0.48) | 83.94 |
| **Lung nodule/mass** | 21 (0.57) | 90 (2.43) | 33 (0.89) | 3561(96.11) | 0.24 (0.15,0.33) | 96.68 |

Abbreviation: LRs, local radiologists; TB, tuberculosis.

**Supplementary Table 3. Local radiologists’ acceptance for the inconsistent results between local radiologists’ initial reading and JF CXR-1 v3.0 at the on-site evaluation phase.**

|  | **LRs+/ JF CXR-1 v3.0-** | **Proportion, n/N (%)*** | **LRs-/ JF CXR-1 v3.0+** | **Proportion, n/N (%)*** |
| --- | --- | --- | --- | --- |
| **Active TB** | 9 | 5/9 (55.56) | 226 | 30/226 (13.27) |
| **Prior TB** | 16 | 3/16 (18.75) | 681 | 86/681 (12.63) |
| **Pneumonia** | 244 | 41/244 (16.85) | 351 | 40/351 (11.40) |
| **Lung nodule/mass** | 33 | 5/33 (15.15) | 90 | 8/90 (8.89) |

Abbreviation: LRs, local radiologists; TB, tuberculosis.

*When LRs’ and JF CXR-1 v3.0’s diagnosis are inconsistent, the proportion of LRs accepting JF CXR-1 v3.0’s diagnosis.
